# The Impact of Operating Ratio on the Static and Fatigue Life of Forward-Acting Rupture Discs

**DOI:** 10.3390/ma18214983

**Published:** 2025-10-31

**Authors:** Haitao Wang, Zhenxi Liu, Honglie Xuan, Hongxin Zhang, Hui Xu, Shan Chen, Jianliang Yu

**Affiliations:** 1 China Special Equipment Inspection and Research Institute, Beijing 100029, China; 2 School of Chemical Engineering, Dalian University of Technology, Dalian 116024, China; 3 CSSC 715th Research Institute, Hangzhou 310023, China; 4 School of Energy and Control Engineering, Changji University, Changji 831100, China; 5 SINOPEC Shanghai Petrochemical Co., Ltd., Shanghai 200540, China

**Keywords:** operating ratio, forward-acting rupture disc, static life, fatigue life, lifespan prediction

## Abstract

Rupture discs are critical safety devices for pressure vessels, yet defining replacement intervals for discs that have not ruptured remains challenging due to limited quantitative life-prediction methods. This study investigates forward-acting rupture discs made of 316 L stainless steel and Inconel 600 under three test conditions: low pressure at room temperature, low pressure at elevated temperature, and ultra-high pressure at elevated temperature. Static hold life and fatigue life were measured over a range of operating ratios *R* = *P*_w_/*P*_b_. To model life–ratio relationships while avoiding far-reaching extrapolation, static life was fitted with a log-normal accelerated-life (AFT) model and fatigue life with a Basquin relation following ASTM E739, reporting 95% prediction bands. Predictions were restricted to validated domains (static: *R* ≥ 0.86) and truncated at five times the groupwise maximum observed life/cycles. Results show a consistent trend for both materials and all conditions: life decreases as *R* increases, with steep sensitivities within the observed range. At matched *R*, Inconel 600 generally exhibits longer life than 316 L. Qualitative failure analysis under constant and cyclic loading indicates progressive plastic deformation, local thinning, and a concomitant reduction in bursting pressure until failure. The proposed in-range predictive framework provides actionable guidance for determining conservative replacement intervals for rupture discs.

## 1. Introduction

A pressure vessel is a closed vessel that contains a gas or liquid and is subjected to internal pressure. Because of demanding service conditions, pressure vessels are susceptible to overpressure incidents [1]. To prevent explosions caused by overpressure, overpressure-relief devices—such as rupture discs and safety valves—are employed as the final line of protection [2]. Rupture discs are essential safety devices used to prevent overpressure in various industries, including petroleum, chemicals, and natural gas production [3]. These simple yet effective devices operate by rupturing when the internal pressure exceeds a predefined threshold, thus protecting pressure vessels, pipelines, and other containment systems from catastrophic failure [3,4,5,6,7]. The primary types of rupture discs include forward-acting, reverse-acting, and flat types, with forward-acting discs being particularly noted for their good fatigue life and rapid response time under pressure fluctuations [8].

The operating principle of a rupture disc involves a pressure differential across the disc, and when this differential reaches a certain threshold, the disc bursts, releasing the built-up pressure [9]. However, for discs that have not been activated over a long period of use there are currently no clear guidelines or regulations regarding whether they should be replaced or continued in service [5]. As a result, studying the lifespan of rupture discs—comprising both static life and fatigue life—is critical to understanding their performance and ensuring continued safety in industrial applications [6].

Static life refers to the time from the application of pressure until rupture under a constant pressure, while fatigue life pertains to the number of loading cycles a rupture disc can withstand before failure under cyclic loading conditions. A key concern is that the premature rupture of a disc is directly linked to its static and fatigue lifespans, which, if poorly understood, may lead to failures below the nominal burst pressure, posing a risk to operational safety [10,11,12]. In particular, rupture discs are vulnerable to damage from cyclic pressure loading, which accelerates material fatigue over time and leads to potential failure even before the maximum burst pressure is reached. In pressure vessels where the internal pressure fluctuates, rupture discs can be subjected to long-term cyclic loading and may fail by fatigue at pressures below their design burst pressure—failures that are not easily detected [13,14]. Fretting-fatigue experiments show that the superposition of normal contact load and cyclic axial load at interfaces precipitates early micro-crack initiation and accelerates crack growth, underscoring the need to account for fatigue damage in service-life assessments [15]. Research on rupture disc fatigue life remains at an early stage with few reported studies; therefore, dedicated fatigue testing is needed to ensure reliability.

While the operating ratio (the ratio of the working pressure to the minimum burst pressure of the disc) is an important factor influencing the lifespan, there are few studies that focus on the quantitative effects of different operating ratios on rupture disc lifespan. International standards such as ASME Boiler and Pressure Vessel Code [16] provides maximum recommended operating ratios for rupture discs, but the actual operating ratio must remain below these limits to ensure optimal lifespan. Exceeding the recommended limits significantly shortens the disc’s service life [17].

There is a lack of comprehensive research on the impact of low and ultra-high-pressure conditions on the lifespan of rupture discs, particularly for ultra-high-pressure systems where pressures can exceed 300 MPa. Low-pressure rupture discs, typically used in storage tanks and pipelines for gases and liquids, are subjected to pressures below 1.6 MPa, whereas ultra-high-pressure rupture discs are used in applications such as high-pressure polyethylene (HPPE) reactors and supercritical fluid systems. These discs require more stringent design considerations, including increased material thickness, enhanced clamping force, and more precise manufacturing tolerances to withstand extreme pressures. Research comparing the lifespan of rupture discs under low-pressure and ultra-high-pressure conditions is still scarce, with little investigation into whether the low-pressure lifespan can accurately represent that under ultra-high pressure [18,19].

This study addresses the gap through two contributions. First, long-duration test platforms for forward-acting rupture discs are developed and validated at low pressure and ultra-high pressure using 316 L stainless steel and Inconel 600. Static hold and fatigue tests were conducted across operating ratios (*R* = *P*_w_/*P*_b_) and temperatures (ambient low pressure, elevated-temperature low pressure, and elevated-temperature ultra-high pressure). Second, using these data, in-range fatigue-life models in the log domain (Basquin-type) are established with quantified uncertainty (95% prediction bands) within validated domains, providing a conservative basis for service-life estimation and replacement-cycle planning.

## 2. Experimental Procedure

### 2.1. Rupture Disc Parameters

**Figure 1:**
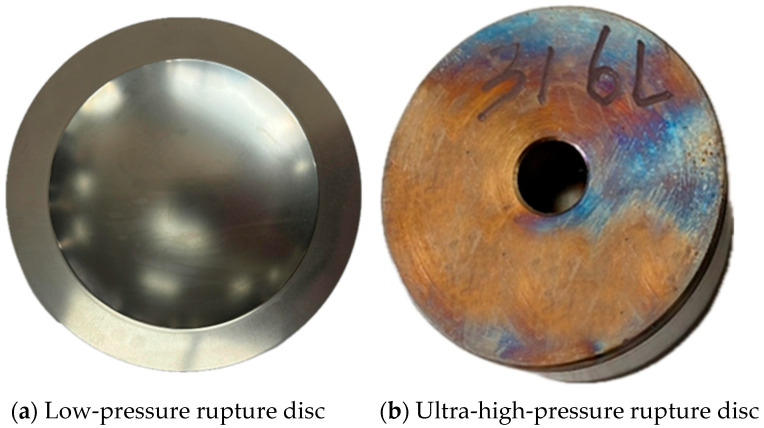
Real image of the rupture disc.

Forward-acting rupture discs are simple to manufacture, cost-effective, and suitable for high-pressure applications, such as large oil storage tanks containing substantial amounts of liquid. The materials commonly used for rupture discs include 316 L and Inconel 600. 316 L stainless steel contains significant amounts of Ni and Cr, resulting in high strength, hardness, and plasticity [20]. Inconel 600 is a Ni-Cr-Fe solid-solution strengthened corrosion-resistant alloy, exhibiting excellent oxidation resistance, corrosion resistance, and superior mechanical properties at high temperatures [21]. In this study, rupture disc materials are 316 L and Inconel 600, with low-pressure and ultra-high-pressure rupture disc images shown in Figure 1.

**Figure 2:**
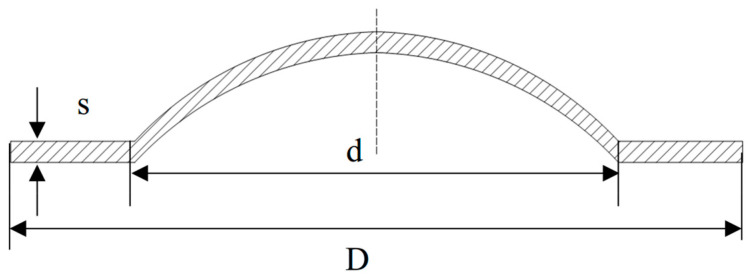
Geometric dimensions of the rupture disc.

The lifespans of rupture discs under low- and ultra-high-pressure conditions may differ [22]. Therefore, static and fatigue tests were conducted on 316 L and Inconel 600 rupture discs at room temperature and high temperature under low pressure. For ultra-high-pressure conditions, only static life tests were conducted. The parameters of the rupture discs are detailed in Table 1, and the geometric schematic diagram is shown in Figure 2, with the geometric dimensions provided in Table 2. The material composition is presented in Table 3 and Table 4. The mechanical property parameters used in this study were obtained in-house from quasi-static tensile tests on coupons cut from the same batch as the rupture discs and are summarized in Table 5. The testing conditions are outlined in Table 6.

The low-pressure rupture discs used in this study were designed with nominal burst pressures below 1.6 MPa, which is consistent with common classification criteria in pressure relief applications (e.g., storage tanks and low-pressure gas systems). In contrast, the ultra-high-pressure rupture discs were developed based on the operational conditions of an actual industrial test platform, where the working pressure exceeds 140 MPa.

Regarding temperature conditions, we selected ambient temperature (20 °C) and high temperature (150 °C) to reflect typical working environments in the chemical and petrochemical industry. The 150 °C condition was chosen to assess the impact of elevated temperature on disc lifetime, particularly related to thermal stress relaxation and material degradation. The temperature gap between ambient and 150 °C allows for a clear comparative analysis of thermal effects on rupture disc durability.

### 2.2. Testing Platforms

This study primarily employs experimental methods to investigate the lifespan of rupture discs under complex working conditions, including room temperature and low pressure, high temperature and low pressure, and high temperature and ultra-high pressure. Three testing platforms were established to accommodate these conditions. The operating ratios applied in this study ranged from 0.85 to 1.00, focusing on high-ratio conditions to simulate near-limit service environments and ensure practical test durations [23].

**Figure 3:**
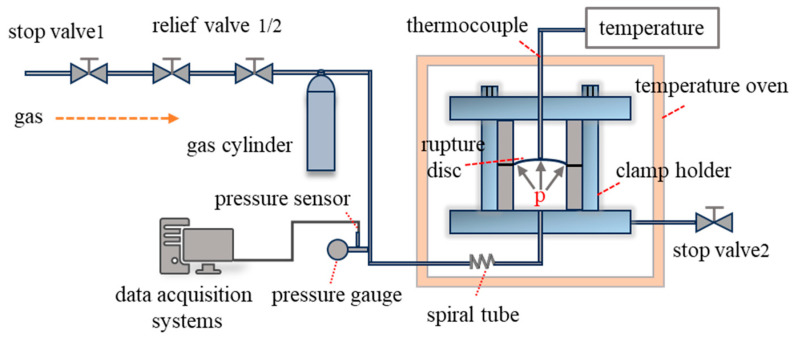
Schematic of the high-temperature static load testing platform.

The static load testing platform for rupture discs includes a gas source, buffer gas cylinder, rupture disc and clamping device, data acquisition system, pressure gauge, sensors, pressure regulator, and shut-off valve. The high-temperature conditions add a high-temperature oven, thermocouples, spiral tubes, and temperature displays compared to the room temperature setup. A schematic of the high-temperature testing platform is shown in Figure 3. The spiral tube is designed to increase the heat exchange area of the circulating gas, ensuring uniform temperature rise within the device. The testing apparatus for ultra-high-pressure static load tests is similar to that for low pressure, with pressure applied using a high-pressure manual pump [24].

**Figure 4:**
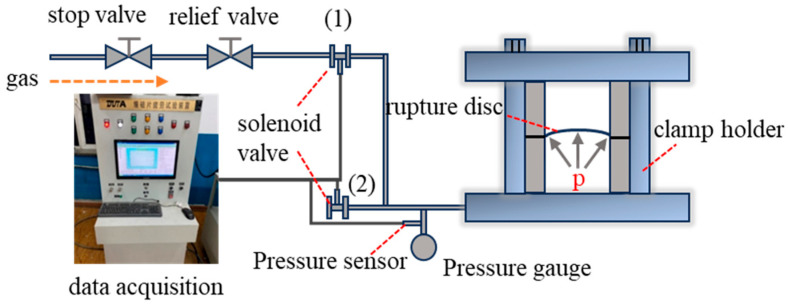
Schematic of the fatigue testing platform. ((1) and (2) are solenoid valves that control the gas inflow and outflow).

**Figure 5:**
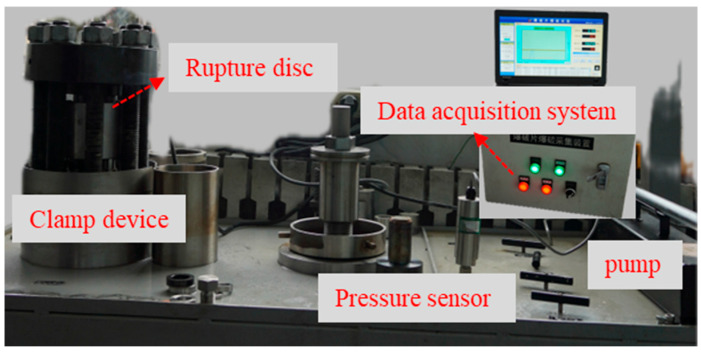
Ultra-high-pressure rupture disc test rig.

The fatigue testing platform for rupture discs consists of a gas source, rupture disc and clamping device, fatigue testing system (data acquisition and control system), pressure gauge, sensors, pressure regulator, shut-off valve, and solenoid valve. The schematic diagram of the fatigue test platform is shown in Figure 4. The pressurization and depressurization processes are automatically controlled by the fatigue testing system, while the pressurization and depressurization rates are controlled manually. The ultra-high-pressure testing platform primarily consists of a flange, rupture disc, clamping device, pressure sensor, thermocouple, temperature control heating coil, ultra-high-pressure pump, and data acquisition system. This ultra-high-pressure device is capable of conducting rupture tests at both room temperature and high temperature under ultra-high-pressure conditions. The ultra-high-pressure rupture disc test rig is shown in Figure 5.

Testing-method enhancements: The test rig incorporates three practical innovations aimed at stable loading, realistic temperature control, and long-duration data capture. First, the inlet line employs a shut-off valve followed by two-stage pressure-reducing valves to set the upstream pressure, which minimizes ripple/overshoot and enables precise control of the operating ratio *R* during both static-hold and fatigue protocols. Second, a thermocouple affixed to the disc crown is continuously monitored by computer, so that the true operating temperature at the specimen—rather than only the chamber temperature—is regulated, better reflecting service conditions. Third, a continuous data-acquisition system records pressure, temperature, and cycle count over the full test duration, allowing month-scale static holds and high-cycle fatigue to be analyzed with prediction bands and lower-quantile metrics. Compared with common single-stage/ambient-set rigs with limited logging, this configuration improves load stability, thermal representativeness, and data completeness.

### 2.3. Testing Procedure

#### 2.3.1. Static Load Testing

First, the gas source was opened to introduce 85% of the burst pressure into the rupture disc, maintaining pressure for 15 min to test the airtightness of the device. After confirming airtightness, stop valve 2 was closed, the gas source was opened, and stop valve 1 was opened. The gas source pressure was initially reduced from 25 MPa to 3 MPa using relief valve 1, and then further adjusted to the required pressure using relief valve 2. Since the burst pressure of the rupture disc was relatively low, minor fluctuations in the gas source can directly alter the operating ratio, affecting the accuracy of the burst pressure. Therefore, a buffer gas cylinder was placed between the relief valve 2 and the rupture disc to increase the gas cavity of the test system, ensuring stable gas pressure acts on the rupture disc surface. The pressure gauge monitors the pressure inside the rupture disc cavity, while the pressure was recorded at regular intervals until the rupture disc ruptures. After bursting, the burst pressure was determined, and the rupture disc was replaced. The process was repeated until the preset number of tests is completed.

#### 2.3.2. Fatigue Load Testing

Before the experiment, the working parameters were set on the fatigue testing machine, and the pressure rise rate was controlled via an external adjusting valve. To avoid excessive pressure rise leading to an actual operating ratio higher than the set ratio, which would reduce fatigue life, the pressure rise rate was set at 0.04 MPa/s. During the experiment, the fatigue testing machine automatically recorded the pressure–time variations in the rupture disc for each cycle. When the rupture disc failed after a certain period, the fatigue testing machine stopped, recording the number of cycles at that moment as the fatigue life of the rupture disc.

**Figure 6:**
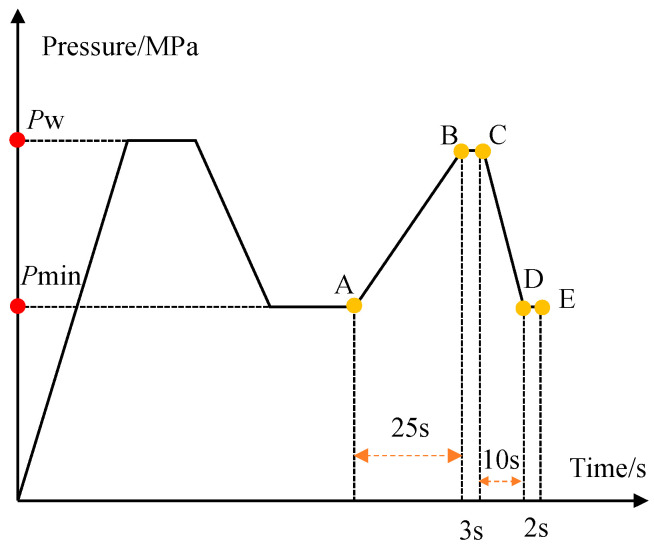
Schematic diagram of fatigue load application.

First, after opening the stop valve, the gas source was introduced into the buffer tank. The pressure continuously increased, and when the pressure sensor indicated that the pressure reached the specified upper limit for the test, the inlet solenoid valve closed, maintaining that pressure. Then, the outlet solenoid valve opened, allowing the rupture disc to depressurize, after which the outlet solenoid valve closed, and the pressure was held for a period. The process from minimum pressure to peak pressure lasted 25 s; after reaching the peak pressure, it was held for 3 s before decreasing to the minimum pressure over 10 s. The minimum pressure was held for 2 s. This constitutes a single fatigue loading cycle, as shown by the curve A-B-C-D-E in the figure, with a total cumulative time of 40 s. This asymmetric cycle was chosen to replicate typical industrial pressure variations in rupture disc systems, rather than idealized symmetric fatigue wave forms. The loading schematic is illustrated in Figure 6. *P*_min_ is the minimum pressure during the fatigue test, set at 0.2 MPa; *P*_w_ is the peak pressure, selected based on different operating ratios.

## 3. Results and Discussion

### 3.1. Verification of Experimental Platform Reliability

The static and fatigue testing platform for rupture discs was constructed using a bolt-flange method to secure the disc holder (Method 1). This method was used to conduct tests on the rupture discs under both static and cyclic loading conditions. On the other hand, the factory testing of the rupture discs employed a hydraulic press (Method 2) to grip the discs for burst pressure calibration.

To determine the consistency of burst pressures obtained from the two methods and to minimize experimental errors caused by differences in gripping devices, it is necessary to validate the reliability of this testing platform through experiments [25]. The comparison results of burst pressures obtained from the two gripping methods are shown in Table 7.

The average bursting pressure of the three groups (1, 2, 3) of 316 L forward-acting rupture discs built in this study is 1.24 MPa, with a deviation of 2.2% from the factory-calibrated bursting pressure of 1.21 MPa, which is within the acceptable range. For the three groups of Inconel 600 rupture discs, the average bursting pressure is 1.08 MPa, with a deviation of 3.5% from the factory-calibrated bursting pressure of 1.04 MPa, also within the acceptable range. It should be noted that the three groups tested for both 316 L and Inconel 600 rupture discs were tested under the same experimental conditions. Therefore, the bursting pressure obtained using the holding device in this test platform shows an acceptable deviation from the calibrated bursting pressure, demonstrating the reliability of the platform. The calibrated bursting pressures for 316 L and Inconel 600 materials are taken as 1.24 MPa and 1.08 MPa, respectively.

### 3.2. Static Load Life

**Figure 7:**
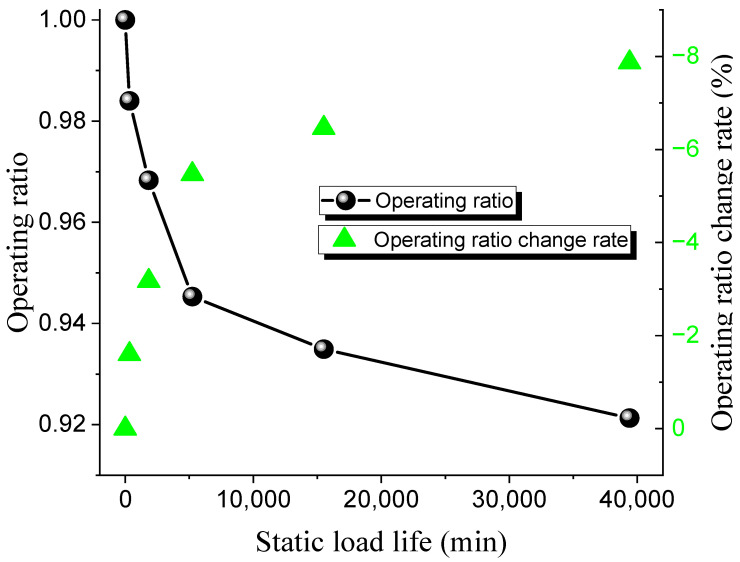
Static load life of 316 L rupture disc at ambient temperature.

**Figure 8:**
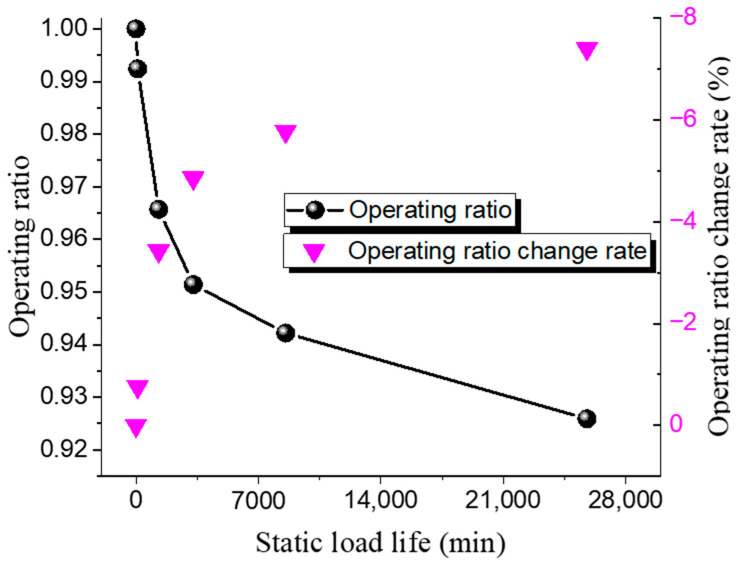
Static load life of 316 L rupture disc at high temperature.

Different operating ratios are selected to conduct bursting tests on 316 L and Inconel 600 forward-acting rupture discs under both ambient and 150 °C high-temperature conditions. The static life test results of the 316 L low-pressure rupture disc at room temperature and high temperature are shown in Table 8 and Table 9, while the relationship between the operating ratio and lifetime is presented in Figure 7 and Figure 8. The rate at which the operating ratio changes between consecutive measurements, calculated as the relative change between the current and previous operating ratios.

The results indicate that the static life of the 316 L rupture disc decreases gradually with an increase in the operating ratio. Under ambient conditions, when the operating ratio decreases from 0.98 to 0.97, the static hold time increases from 335 min to 1840 min. Similarly, when the operating ratio decreases from 0.93 to 0.92, the static hold time increases from 15,510 min to 39,400 min. The pattern of static life under high-temperature conditions is similar to that under ambient conditions. At high operating ratios, when the operating ratio decreases from 0.99 to 0.97 (a decrease of 0.03), the static hold time increases from 96 min to 1301 min. When the operating ratio decreases from 0.94 to 0.93 (a decrease of 0.016), the static hold time increases from 8559 min to 25,780 min. These results suggest that the static life of the rupture disc is significantly affected by changes in the operating ratio.

**Figure 9:**
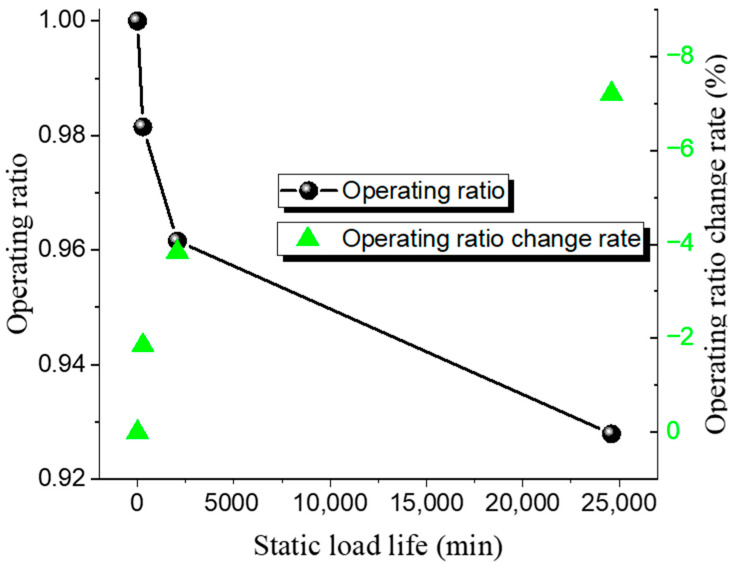
Static load life of Inconel 600 rupture disc at ambient temperature.

**Figure 10:**
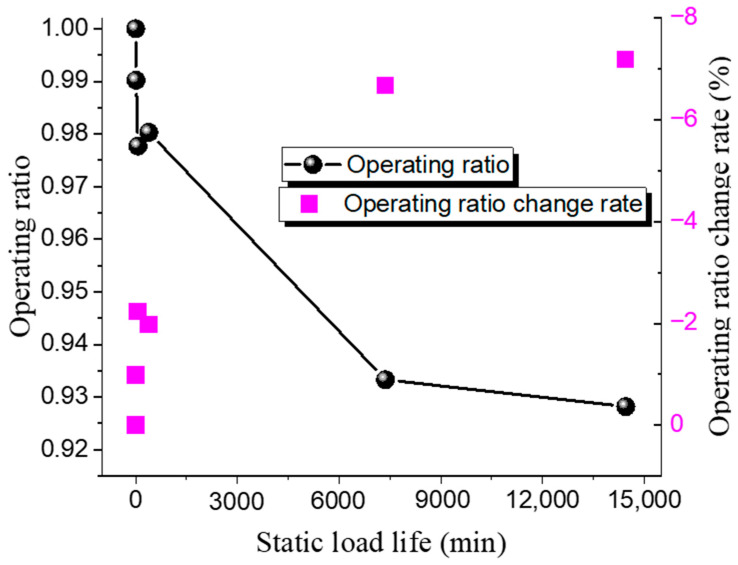
Static load life of Inconel 600 rupture disc at high temperature.

The static life test results of the Inconel 600 low-pressure rupture disc at room temperature and high temperature are shown in Table 10 and Table 11, while the relationship between the operating ratio and lifetime is presented in Figure 9 and Figure 10. Similarly to the 316 L disc, its static life decreases with an increase in the operating ratio. Under ambient conditions, when the operating ratio decreases from 0.98 to 0.96 (a decrease of 0.02), the static hold time increases from 280 min to 2075 min. At an operating ratio of 0.93, the static hold time is 24,600 min under ambient conditions and 14,450 min under high-temperature conditions.

**Figure 11:**
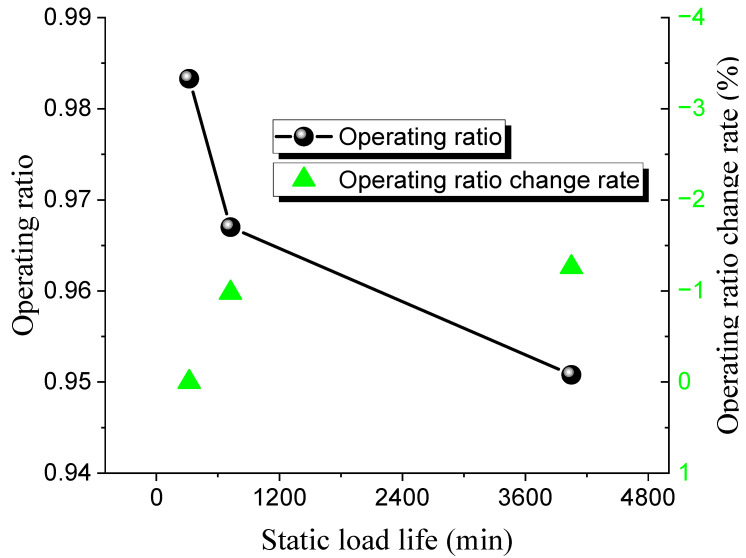
Static load life of 316 L ultra-high-pressure rupture disc at high temperature.

**Figure 12:**
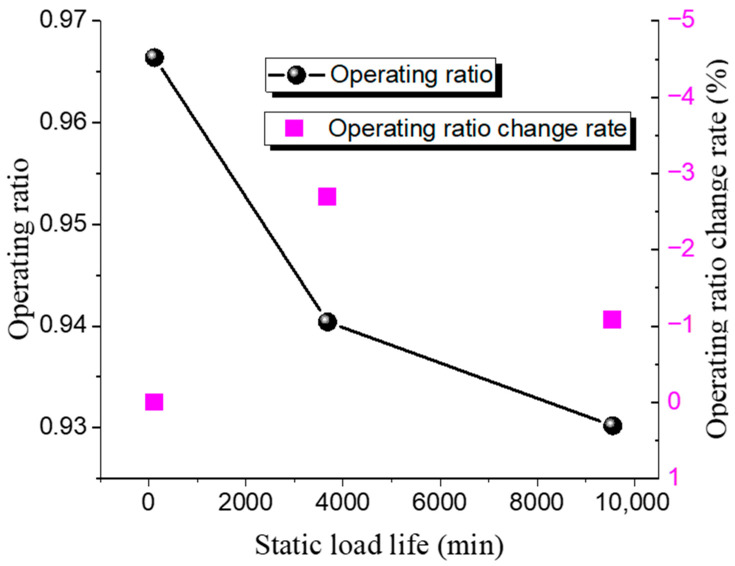
Static load life of Inconel 600 ultra-high-pressure rupture disc at high temperature.

The static lifespan tests for 316 L and Inconel 600 ultra-high-pressure rupture discs at different operating ratios were conducted. The test results are shown in Table 12 and Table 13, while the relationship between the operating ratio and lifetime is presented in Figure 11 and Figure 12.

The static burst pressure for the 316 L rupture disc is 121 MPa, with a maximum operating ratio of 0.98 and a minimum of 0.95. For the Inconel 600 rupture disc, the static burst pressure is 146 MPa, with a maximum operating ratio of 0.97 and a minimum of 0.93. The pattern observed is consistent with that of low-pressure rupture discs; as the operating ratio increases, the static life of the ultra-high-pressure rupture discs decreases. Under the same operating ratio, the static life of the 316 L discs is higher than that of the Inconel 600 discs.

### 3.3. Fatigue Life

As the operating ratio decreases, the number of fatigue cycles increase, making the tests more challenging. A fatigue cycle refers to the number of loading and unloading cycles that the rupture disc undergoes during the fatigue testing. As the operating ratio decreases, it requires more cycles to reach failure, leading to longer testing durations. In this study, the lower limit for the operating ratio in the rupture disc fatigue experiments is set at 0.85. The fatigue life test results for the 316 L low-pressure rupture disc are shown in Table 14, while the fatigue test results for the Inconel 600 low-pressure rupture disc are shown in Table 15.

**Figure 13:**
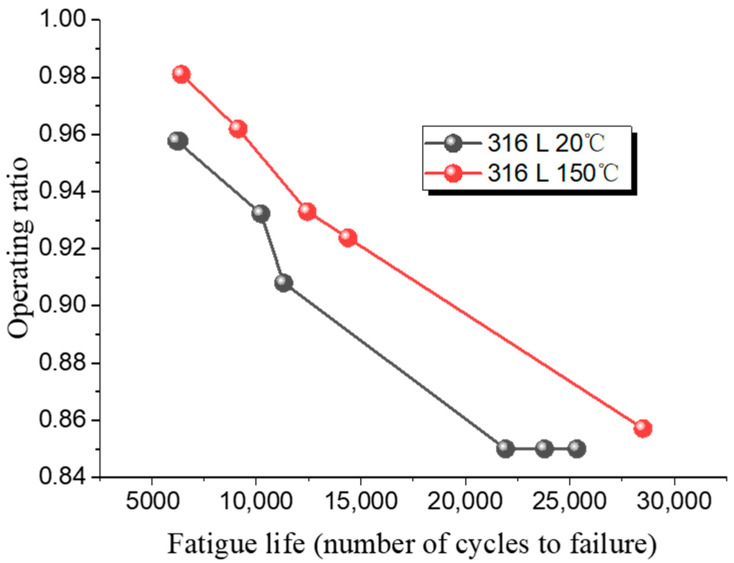
Fatigue life of 316 L low-pressure rupture disc at ambient and high temperatures.

Figure 13 shows the fatigue test results for 316 L at room and high temperatures. As the operating ratio increases, the number of cycles required for fatigue failure of rupture discs made from both materials gradually decreases.

**Figure 14:**
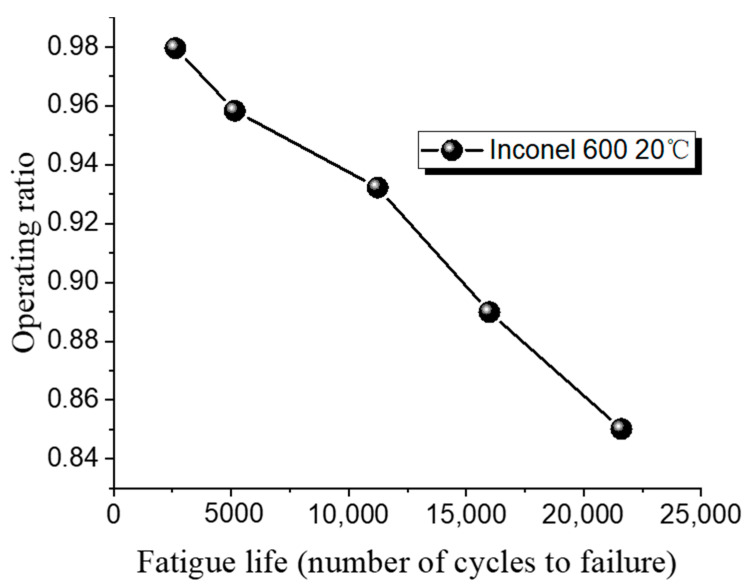
Fatigue life of Inconel 600 low-pressure rupture disc at ambient temperature.

At a low operating ratio of 0.85, the 316 L rupture disc fails after 21,930 cycles. At a higher operating ratio of 0.96, failure occurs after only 6306 cycles, indicating a reduction of 15,624 cycles with a 0.11 change in operating ratio. When the operating ratio is 0.96, the fatigue life of 316 L at high temperature is 9139 cycles, and it increases to 12,446 cycles when the operating ratio drops to 0.93. Figure 14 shows the fatigue test results for Inconel 600 at room temperature. For Inconel 600, increasing the operating ratio from 0.85 to 0.93 results in a reduction of cycles from 21,607 to 11,233, a difference of 10,374 cycles.

These results indicate that changes in the operating ratio have a greater impact on fatigue cycles at lower ratios. Additionally, under the same operating ratio, the fatigue life of rupture discs at high temperature is superior to that at room temperature.

## 4. Life Prediction

### 4.1. Static Load Life Prediction

To quantify the effect of the operating ratio *R* = *P*_w_/*P*_b_ on the static hold life *T*, we adopt a log-normal accelerated-life (AFT) model:

(1) $$\ln T=a+b\ln R+\varepsilon ,\;\varepsilon \sim N(0,s^{2}).$$

The parameters (*a*, *b*) are estimated by ordinary least squares, and the residual standard deviation is $s=\sqrt{SSE/(n-2)}$. For any target ratio R* (let x* = lnR*), the 95% prediction band for a new observation in the log domain is

(2) $$y_{PI}^{\pm }(x^{*})=\hat{y}(x^{*})\pm t_{0.975,n-2}\hat{s}\sqrt{1+\frac{1}{n}+\frac{{\left(x^{*}-\bar{x}\right)}^{2}}{S_{xx}}}$$

(3) $$y_{5\%}(x^{*})=\hat{y}(x^{*})+t_{0.05,n-2}\hat{s}\sqrt{1+\frac{1}{n}+\frac{{\left(x^{*}-\bar{x}\right)}^{2}}{S_{xx}}}$$

where $\hat{y}(x^{*})=\hat{a}+\hat{b}x^{*}$, $\bar{x}=\frac{1}{n}\sum \ln R_{i}$, and $S_{xx}=\sum {\left(\ln R_{i}-\bar{x}\right)}^{2}$. All bands are computed in the log domain and then exponentiated to obtain lifetimes in minutes.

To avoid non-scientific conclusions from far-reaching extrapolation, predictions are reported only within the validated domain *R* ≥ 0.86 and are truncated at 5*T*_max_ (five times the groupwise maximum observed life). Groups with fewer than four data points (*n* = 3) are not used for quantitative prediction.

**Figure 15:**
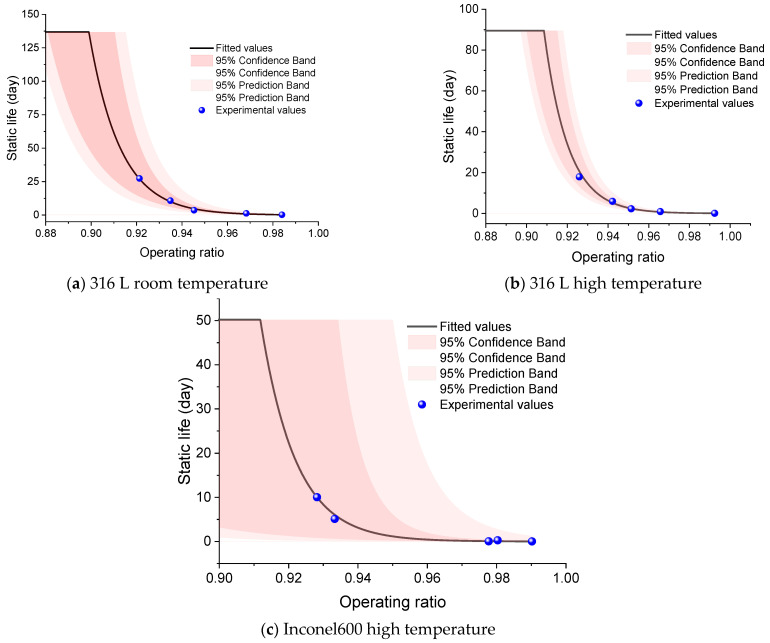
Static life prediction.

Based on the three datasets—316 L at room temperature, 316 L at elevated temperature, and Inconel 600 at elevated temperature (each with *n* = 5)—the fitted curves with 95% prediction bands are shown in Figure 15. Table 16 summarizes the fitted coefficients for the static-life model. The magnitude ∣*b*∣ quantifies the slope—larger ∣*b*∣ indicates a steeper life–ratio trend (greater sensitivity to *R*). For 316 L, at a given operating ratio *R*, the static life at elevated temperature exceeds that at room temperature. In high-temperature conditions, the static life of the rupture discs exceed those at ambient temperature due to two main factors: first, the elimination of residual stresses at high temperatures enhances the disc’s load-bearing capacity, thus increasing its life. Second, analysis of fracture surfaces shows that at elevated temperatures, the disc experiences significant deformation, indicating a faster thickness reduction and weakened load capacity, making failure more likely [26].

### 4.2. Fatigue Life Prediction

To quantify the effect of the operating ratio *R* = *P*_w_/*P*_b_ on the fatigue life *N* of rupture discs, we employ the Basquin relation and perform regression and interval estimation following ASTM E739 [27].

(4) $$y=\log_{10}N=a+bx+\epsilon$$

(5) $$x=\log_{10}R$$

Equations (4) and (5) describe the relationship between the operating ratio and the fatigue life of the rupture disc. These equations are derived based on the log-normal distribution for fatigue life. The parameters a and b are the fit coefficients that characterize the sensitivity of fatigue life to the operating ratio. To avoid unreliable conclusions from far-reaching extrapolation, predictions are reported only within a validated domain: for 316 L, *R* ≥ 0.8; for Inconel groups, predictions are confined to the vicinity of each group’s observed lower bound and not below 0.8. Predicted cycle counts are truncated at 5*N*_max_ (five times the groupwise maximum observed cycles), and values beyond this cap are not interpreted. Groups with only *n* = 3 data points are not used for quantitative predictions; only the fitted curve and associated uncertainty are reported.

**Figure 16:**
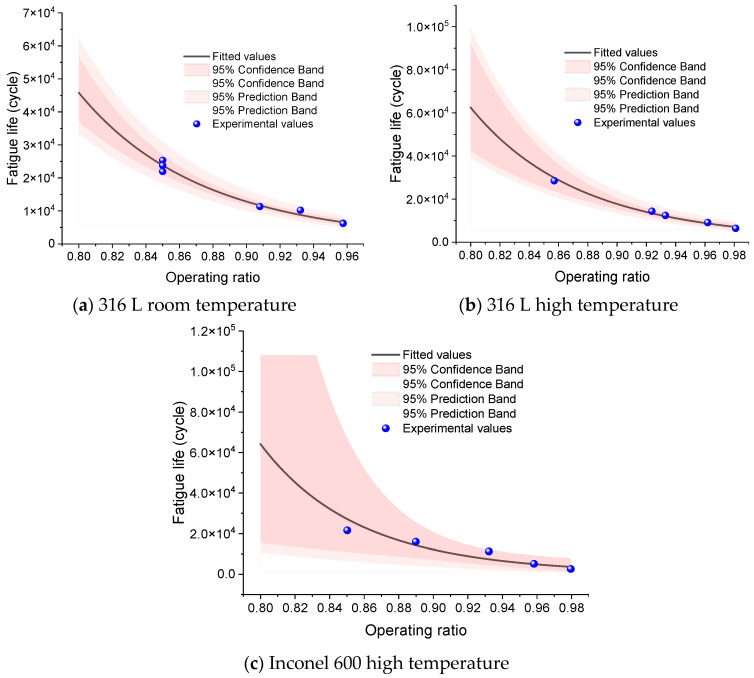
Fatigue life prediction curves for rupture discs.

For the three datasets—316 L at room temperature, 316 L at elevated temperature, and Inconel-600 at elevated temperature (each with *n* = 5)—the Basquin fits with 95% prediction bands are shown in Figure 16a–c. Table 17 presents the fitted coefficients for the fatigue-life model. The sensitivity to the operating ratio is high when ∣*b*∣ is large, indicating a steep sensitivity of life to *R*. For 316 L, when extending the validated domain down to *R* = 0.8, both the point estimates and the upper bounds of the prediction bands remain below 5*N*_max_, satisfying the truncation criterion; hence quantitative predictions are reported for *R* ∈ [0.8, *R*_max_]. The Inconel-600 (elevated temperature) group shows larger residual scatter (higher s); for engineering use, we recommend the 5% quantile life *N*_5%_ as a conservative estimate. Groups with *n* = 3 (e.g., 316 L under ultra-high pressure, and Inconel at room temperature/elevated-temperature ultra-high pressure) are not used for numerical predictions due to very low statistical degrees of freedom [28].

## 5. Conclusions

(1) For forward-acting rupture discs, both static and fatigue life decrease monotonically with increasing operating ratio *R*. Within the validated domains (static *R* ≥ 0.86; fatigue *R* ≥ 0.8, the fitted AFT/Basquin models with 95% prediction bands enable conservative, quantitative life estimates suitable for maintenance planning.

(2) Across conditions, Inconel 600 generally outperforms 316 L at the same *R* (longer life), while retaining the same qualitative trend of decreasing life with increasing *R*. The models capture the high sensitivity of life to small changes in *R* near the bursting-pressure limit.

(3) Under both constant and cyclic loading, progressive deformation leads to local thickness reduction and a decline in bursting pressure, culminating in failure—consistent with the observed life–ratio behavior.

(4) Observations for ultra-high-pressure discs follow the same qualitative pattern as those at low pressure, suggesting that methodologies and trends established at low pressure are informative for ultra-high-pressure life studies.

In future work, life tests will be conducted at lower operating ratios (beyond the present validated domain) to directly characterize the long-life regime and narrow the prediction bands. In parallel, an elastoplastic finite-element model, calibrated to our in-house tensile data, will be developed to map the operating ratio R to local strain/strain range and thereby refine life prediction.

## Figures and Tables

**Table 1:** Rupture disc parameters.

Parameters	316 L		Inconel 600	
	Low Pressure	Ultra-High Pressure	Low Pressure	Ultra-High Pressure
Design Burst Pressure/MPa	1.0	140	1.0	160
Burst Pressure Tolerance	±3	±4	±3	±4
Test Burst Pressure (Room Temperature 20 °C)/MPa	1.24	142	1.08	159
Test Burst Pressure (High Temperature 150 °C)/MPa	1.06	121	1.02	146

**Table 2:** Geometric dimensions.

Material	Pressure Level	Membrane Thickness/mm	Burst Diameter/mm	Outer Diameter/mm
316 L	Low pressure	0.05	91	115
316 L	Ultra-high pressure	0.5	10	42
Inconel 600	Low pressure	0.05	91	115
Inconel 600	Ultra-high pressure	0.8	15	42

**Table 3:** Material composition of 316 L/% (mass fraction).

C	Si	Mn	P	S	Cr	Ni	Mo	N
0.0162	0.0618	0.1354	0.0283	0.0014	16.52	10.05	2.03	0.0106

**Table 4:** Material composition of Inconel 600/% (mass fraction).

C	Mn	P	S	Si	Cr	Ni	Cu	Fe
0.05	0.24	0.009	0.0002	0.19	15.81	74.80	0.03	8.26

**Table 5:** Mechanical property parameters.

Material	Yield Strength/MPa	Ultimate Strength/MPa	Elongation/%
316 L	263	615	58
Inconel 600	351	703	38

**Table 6:** Testing conditions.

Test Type	Low Pressure Conditions		Ultra High-Pressure Conditions	
	Material	Temperature/°C	Material	Temperature/°C
Static Life	316 L	20	316 L	150
		150	Inconel 600	150
	Inconel 600	20	——	
		150		
Fatigue Life	316 L	20	——	
		150		
	Inconel 600	20		

**Table 7:** Comparison of burst pressures from two rupture disk holding devices.

Number	Material	Burst Pressure/MPa	
		Measured Value	Factory Calibration Value
1	316 L	1.24	1.21
2		1.23	1.21
3		1.24	1.21
		Average Value	Deviation from Factory Calibration Value/%
		1.24	2.2
4	Inconel 600	1.08	1.04
5		1.08	1.04
6		1.07	1.04
		Average Value	Deviation from Factory Calibration Value/%
		1.08	3.5

**Table 8:** Static load life of 316 L low-pressure rupture disc at room temperature.

Test	Temperature /°C	Static Load Pressure /MPa	Operating Ratio	Operating Ratio Change Rate /%	Hold Time /min
1	20	1.22	0.984	−1.6	335
2	20	1.20	0.9683	−3.17	1840
3	20	1.17	0.9453	−5.47	5248
4	20	1.16	0.9349	−6.46	15,510
5	20	1.14	0.9213	−7.87	39,400

**Table 9:** Static load life of 316 L low-pressure rupture disc at high temperature.

Test	Temperature /°C	Static Load Pressure /MPa	Operating Ratio	Operating Ratio Change Rate /%	Hold Time /min
1	151	1.052	0.9924	−0.76	96
2	151	1.024	0.9657	−3.43	1301
3	151	1.008	0.9514	−4.86	3284
4	152	0.9987	0.9422	−5.78	8559
5	151	0.9815	0.9259	−7.41	25,780

**Table 10:** Static load life of Inconel 600 low-pressure rupture disc at room temperature.

Test	Temperature /°C	Static Load Pressure /MPa	Operating Ratio	Operating Ratio Change Rate /%	Hold Time /min
1	20	1.08	1	0	0
2	20	1.06	0.9815	−1.85	280
3	20	1.05	0.9616	−3.84	2075
4	20	1.00	0.9279	−7.21	24,600

**Table 11:** Static load life of Inconel 600 low-pressure rupture disc at high temperature.

Test	Temperature /°C	Static Load Pressure /MPa	Operating Ratio	Operating Ratio Change Rate /%	Hold Time /min
1	151	1.01	0.9902	−0.98	13
2	151	0.997	0.9777	−2.23	69
3	150	1.0	0.9803	−1.97	390
4	152	0.952	0.9333	−6.67	7362
5	150	0.9468	0.9282	−7.18	14,450

**Table 12:** Static load life of 316 L ultra-high-pressure rupture disc at high temperature.

Test	Temperature /°C	Static Load Pressure /MPa	Operating Ratio	Operating Ratio Change Rate /%	Hold Time /min
1	152	119	0.9833	0	320
2	152	117	0.967	−0.98	723
3	152	115	0.9508	−1.26	4050

**Table 13:** Static load life of Inconel 600 ultra-high-pressure rupture disc at high temperature.

Test	Temperature /°C	Static Load Pressure /MPa	Operating Ratio	Operating Ratio Change Rate /%	Hold Time /min
1	151	141	0.9664	0	117
2	151	137.3	0.9404	−2.69	3683
3	151	135.8	0.9302	−1.08	9552

**Table 14:** Fatigue life of 316 L low-pressure rupture disc.

Test	Temperature/°C	Valley Pressure/MPa	Peak Pressure/MPa	Operating Ratio	Fatigue Cycles
1	20	0.2	1.19	0.9577	6306
2	20	0.2	1.19	0.9577	6201
3	20	0.2	1.16	0.9322	10,221
4	20	0.2	1.126	0.908	11,316
5	20	0.2	1.05	0.85	21,930
6	20	0.2	1.05	0.85	25,341
7	20	0.2	1.05	0.85	23,795
8	153	0.2	1.03	0.981	6417
9	152	0.2	1.01	0.9619	9139
10	152	0.2	0.98	0.933	12,446
11	150	0.2	0.97	0.9238	14,385
12	150	0.2	0.90	0.8571	28,500

**Table 15:** Fatigue life of Inconel 600 low-pressure rupture disc.

Test	Temperature/°C	Valley Pressure/MPa	Peak Pressure/MPa	Operating Ratio	Fatigue Cycles
1	20	0.2	1.06	0.9796	2630
2	20	0.2	1.035	0.9583	5157
3	20	0.2	1.007	0.9322	11,233
4	20	0.2	0.96	0.8899	15,987
5	20	0.2	0.98	0.8502	21,607

**Table 16:** Fitted coefficients for the static-life model.

	316 L Room Temperature	316 L High Temperature	Inconel 600 High Temperature
*a*	77.61	88.26	101.05
*b*	−72.77	−84.20	−98.55

**Table 17:** Fitted coefficients for the fatigue-life model.

	316 L Room Temperature	316 L High Temperature	Inconel 600 High Temperature
*a*	8.32	8.66	7.91
*b*	−10.79	−10.68	−14.13

## Data Availability

The original contributions presented in this study are included in the article. Further inquiries can be directed to the corresponding authors.

## References

[1] Friedel L. (1988). Two phase pressure drop in a rupture disc/safety valve unit. J. Loss Prev. Process Ind..

[2] Perbal R. (1993). Transient flow phenomena and reaction forces during blowdown of gas at high pressures through relief lines behind rupture discs. Process Saf. Prog..

[3] Mutegi M.K., Schmidt J., Denecke J. (2019). Sizing rupture disk vent line systems for high-velocity gas flows. J. Loss Prev. Process Ind..

[4] Shannak B. (2010). Experimental and theoretical investigation of gas–liquid flow pressure drop across rupture discs. Nucl. Eng. Des..

[5] Yu Q., Hui H., Gong J. (2018). Design Research of the Double Rupture Disc Safety Relief Device Used on the Long Tube Trailer. IOP Conf. Ser. Mater. Sci. Eng..

[6] Chen D., Xu X., Xuan H., Guo B., Huo L., Yu J. (2023). Rupture Disc Monitoring Using Electro-mechanical Impedance (EMI): A Feasibility Study. J. Nondestruct. Eval..

[7] Asahara M., Saburi T., Ando T., Takahashi Y., Miyasaka T., Kubota S. (2021). Self-ignited flame behavior of high-pressure hydrogen release by rupture disk through a long tube. Int. J. Hydrogen Energy.

[8] Gong L., Jin K., Zheng X., Han Y., Yao Y., Duan Q., Zhang Y., Sun J. (2023). Effect of Al-made burst disk on the shock wave and the spontaneous ignition of high-pressure hydrogen during its sudden discharge. J. Energy Storage.

[9] Jeong J.Y., Lee J., Yeom S., Choi W., Kim T.G., Hong S.C., Ryu M., Kim H., Lee S.B. (2012). A study on the grooving process of a cross-scored rupture disc. Int. J. Precis. Eng. Manuf..

[10] Sun B., Li Z. (2014). A multi-scale damage model for fatigue accumulation due to short cracks nucleation and growth. Eng. Fract. Mech..

[11] Santecchia E., Hamouda A.M.S., Musharavati F., Zalnezhad E., Cabibbo M., El Mehtedi M., Spigarelli S. (2016). A Review on Fatigue Life Prediction Methods for Metals. Adv. Mater. Sci. Eng..

[12] Tang J.Q., Geng L.Y., Gong J.M. (2019). Analysis on Bursting of Rupture Disc Made by Inconel 600 Alloy. Key Eng. Mater..

[13] Bhattacharya B., Ellingwood B. (1998). Continuum damage mechanics analysis of fatigue crack initiation. Int. J. Fatigue.

[14] Miller K.J. (1987). The behaviour of short fatigue cracks and their initiation part I—A review of two recent books. Fatigue Fract. Eng. Mater. Struct..

[15] Arora P.R., Jacob M.S.D., Salit M.S., Ahmed E.M., Saleem M., Edi P. (2007). Experimental evaluation of fretting fatigue test apparatus. Int. J. Fatigue.

[16] Sotoodeh K. (2023). The Design of Pressure Safety and Relief Valves for Overpressure Protection: Essential considerations. Trans. Indian Natl. Acad. Eng..

[17] Doelp L.C., Brian P.L.T. (1982). Reliability of pressure protective systems. A Markov analysis. Ind. Eng. Chem. Fundam..

[18] Yang C., Hui H., Huang S. (2020). Theoretical and experimental study on sealing performance of a novel ultra-high pressure bursting disc. Proc. Inst. Mech. Eng. Part E J. Process Mech. Eng..

[19] Rüsenberg S., Vonnahme G. Failure Analysis of High Pressure Rupture Discs and Effective Counter Measures. Proceedings of the ASME 2016 Pressure Vessels and Piping Conference.

[20] Ralls A.M., Menezes P.L. (2024). Revealing the fretting corrosion mechanisms of laser shock peened cold spray 316 L stainless steel. Tribol. Int..

[21] Mishra N.K., Shrivastava A. (2023). Improvement in strength and ductility of rotary friction welded Inconel 600 and stainless steel 316 L with Cu interlayer. CIRP J. Manuf. Sci. Technol..

[22] Yuan D., Li X., Ni M., Zhu J., Wang J., Yu T., Wang J., Shen J. (2025). Theoretical design and experimental validation of rupture discs for water heat pipe blackbody source. Measurement.

[23] Liu Z., Zhou D., Xu H., Luo Y., Yu J., Yan X. (2025). Experimental and numerical investigation of burst characteristics of ultra-high pressure rupture discs. Int. J. Press. Vessel. Pip..

[24] Sun S., Chen P., Zhai X., Liu Y. (2023). Numerical study on the influence mechanism of different types of burst disc on high pressure hydrogen spontaneous combustion in tube. J. Energy Storage.

[25] Liu L., Yuan C., Li W., Li B., Liu X. (2021). Influence of Moulding Pressure on the Burst Pressure of Reverse-Acting Rupture Discs. Processes.

[26] Zhu H., Xu W., Luo Z., Zheng H. (2020). Finite Element Analysis on the Temperature- Dependent Burst Behavior of Domed 316 L Austenitic Stainless Steel Rupture Disc. Metals.

[27] Goedel F., Chamberlain Pravia Z.M., Mezzomo G.P. (2019). Methodology for assessment of statistical planning effects on the S-N curve determination using Monte Carlo simulations. Fatigue Fract. Eng. Mater. Struct..

[28] Chu Z., Lili L., Wei L., Beibei L., Mingxing L. (2024). Effects of fatigue and deformation on bursting pressure of conventional slotted blasting disc. J. Loss Prev. Process Ind..

